# Cervicogenic Dizziness After Self-Manipulation of the Cervical Spine

**DOI:** 10.7759/cureus.37051

**Published:** 2023-04-03

**Authors:** Eric Chun-Pu Chu, Andy Fu Chieh Lin, Gordon Cheung, Kevin Hsu Kai Huang

**Affiliations:** 1 Chiropractic and Physiotherapy Centre, New York Medical Group, Hong Kong, HKG

**Keywords:** cervicogenic dizziness, self spinal manipulation, chiropractor, spinal manipulation, chiropractic therapy

## Abstract

Patients with pre-existing cervical pathologies who experience dizziness and related neck pain are referred to as having cervicogenic dizziness. We describe a case of a 49-year-old female who presented with acute onset of vertigo and imbalance following self-manipulation of the cervical spine. Examination revealed a restricted cervical range of motion, muscle hypertonicity, and positive neurological signs. Radiographs demonstrated loss of normal cervical lordosis. The patient was diagnosed with cervicogenic dizziness and prescribed chiropractic treatments that included spinal manipulation, soft tissue release, and rehabilitative exercises. After four weeks of care, her symptoms had improved. At the six-month follow-up, the patient remained asymptomatic with a full cervical range of motion. This case highlights the risks associated with neck manipulation and the effectiveness of chiropractic treatment for cervicogenic dizziness. Patients should be counseled to seek evaluation and treatment from appropriate medical professionals for neck issues or dizziness/imbalance.

## Introduction

Proprioceptive receptors are highly developed in the cervical spine. The information derived from these receptors is integrated with the information from the visual and vestibular systems of the central nervous system. It provides feedback for the eye and neck muscles and helps facilitate the coordinated motion of the eyes, neck, head, and body through different reflex systems [1]. Cervical proprioceptive dysfunction due to various neck issues can alter orientation in space, leading to a sensation of disequilibrium [2]. Hence, cervical spine pathologies can lead to dizziness [1]. Cervicogenic dizziness (CGD) is a clinical illness that manifests as neck discomfort and dizziness in those who already have cervical diseases [2]. These patients may present with a constellation of symptoms, including loss of balance as well as a sensation of lightheadedness and vertigo, which are exacerbated by specific neck positions or movements [3].

Manipulation of the cervical spine by an untrained individual or "self-manipulation" can lead to adverse outcomes, including damage to cervical tissues. It could also lead to serious complications such as Brown-Sequard syndrome [4], vertebral artery dissection [5], epidural hematoma [6], and herniation of the intervertebral discs [7]. Cervical dysfunction can also manifest as increased neck pain or stiffness and CGD [8]. We performed a literature review using PubMed and Google Scholar on March 15, 2023. Only a few spinal injuries have been reported after self-manipulation of the cervical spine in the literature. However, this case study is the first to highlight the association between CGD and self-manipulation of the cervical spine, when performed by someone other than a trained clinician.

## Case presentation

**Figure 1:**
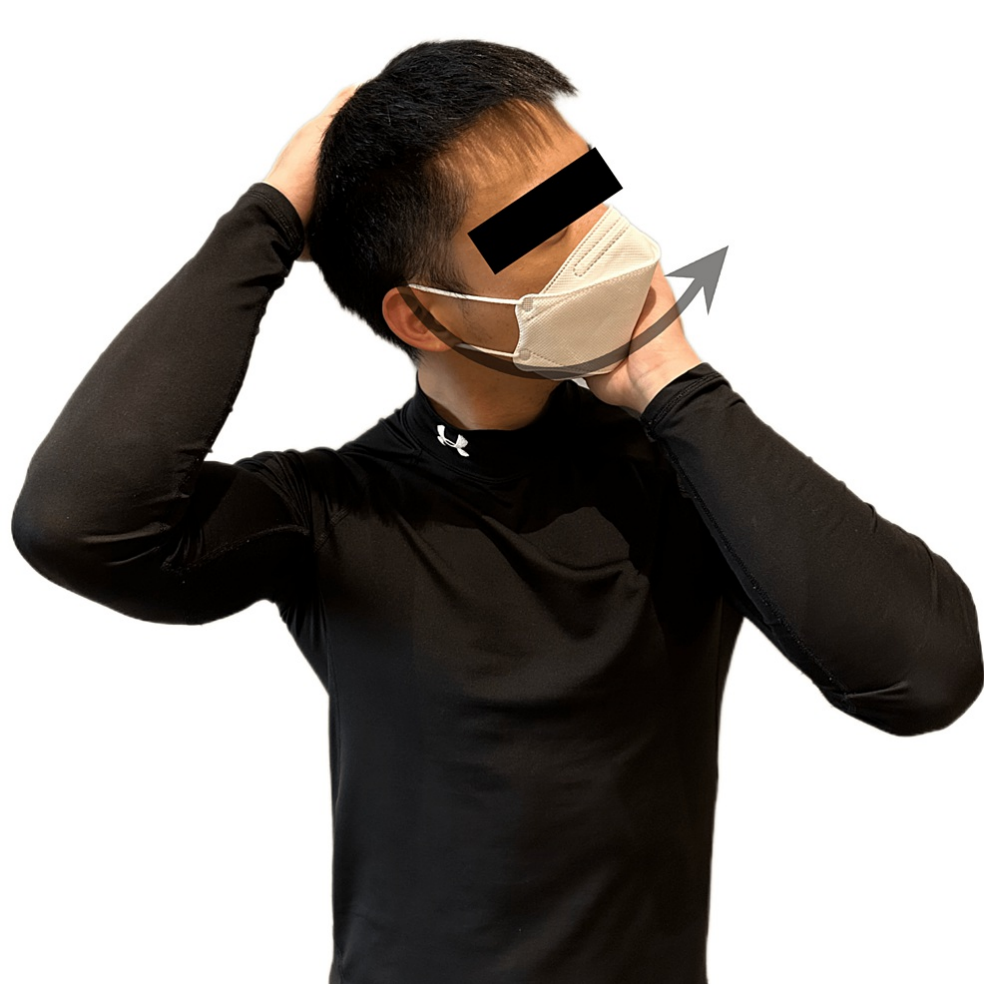
Demonstration of manual self-manipulation of the cervical spine The patient exhibits self-manipulative movement by turning the head left to rotate the cervical spine by tiling the head side to side to laterally flex the cervical spine.

**Figure 2:**
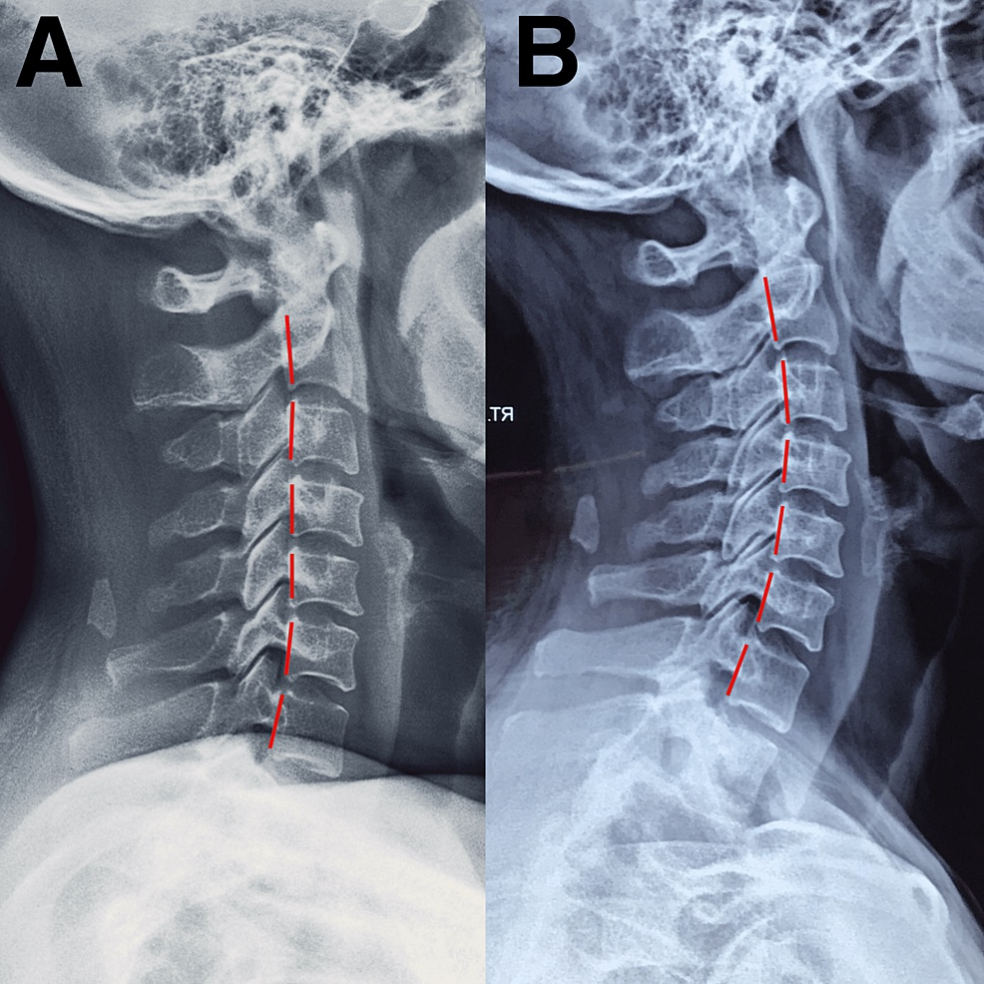
Cervical radiograph in lateral view (A) Cervical alignment demonstrates reduced cervical lordosis. Marginal osteophytes of cervical vertebral bodies and narrowing of C5/C6 disc space were also identified. (B) On the sixth-month re-evaluation, normal bony alignment and articulations were noted.

A 49-year-old female presented with neck pain, acute onset of vertigo, and loss of balance that developed immediately after self-manipulation of her cervical spine. While stretching in the morning, the patient twisted her neck to one side (Figure 1) and suddenly experienced a sensation of the room spinning around her, as well as nausea and unsteadiness on her feet. She also reported recurring episodes of dizziness that resulted in impaired functioning, occupational performance, and sleep quality. The patient admitted to excessively using her mobile phone to watch Netflix shows, typically spending over eight hours per day in a forward-head posture. She also had a history of chronic neck stiffness, and regularly underwent massage therapy to ease neck stiffness and pain. She frequently practiced self-manipulation of her neck to gain temporary relief. She denied any prior history of headaches or dizziness. She visited her primary care physician and underwent radiographs of the cervical spine (Figure 2A). The images demonstrated a loss of normal cervical lordosis with decreased curvature and spacing between the C3 and C7 vertebrae. The patient was referred to a chiropractor for evaluation and treatment.

On initial presentation, the patient exhibited a guarded cervical posture with a limited active range of motion. Her active cervical range of motion was limited to 70°/90° of rotation, 70°/80° of flexion, and 20°/70° of extension. She had palpable hypertonicity in the right sternocleidomastoid, right splenic capitis, and bilateral trapezius muscles. Intersegmental dysfunction was identified at the C3/4 and C4/5 levels. Provocative testing was positive for right-sided neurological symptoms, according to Spurling's test. Bilaterally, the upper and lower extremities' deep tendon reflexes were normal and graded at 2+. Sensory deficits were detected in the right C5 dermatome with concurrent motor weakness C4/5 in the right deltoid and bicep muscles. Her World Health Organization Quality of Life (WHOQOL) score was 64/100, indicating moderate impairment of quality of life. The chiropractor reviewed her cervical radiographs, which demonstrated reduced lordosis and sclerotic changes in the upper endplates of the C5/6 (Figure 2A). The patient was diagnosed with CGD. The chiropractor suspected the cause of the symptoms may be due to her self-manipulation of the cervical spine.

The chiropractic treatment plan was designed to reduce cervical pain and instability, restore active range of motion, and prevent further neurological compromise. Interventions included high-velocity, low-amplitude manipulation of restricted segments, long-axis distraction of segments superior to C5, and post-isometric relaxation to lengthen the hypertonic musculature. The treatment regimen was delivered three times/week for four weeks. It resulted in a 50% reduction in pain and neurological symptoms. The patient regained the ability to maintain an upright cervical posture after the first week. By the 14th day of the treatment, her vertigo and imbalance had fully resolved. The frequency of treatment was reduced to one time/week for the next five months with the addition of alignment correction to reduce residual symptoms. Cervical extension-compression traction (chiropractic biophysics technique) was added to the treatment plan for 15 minutes during each session. The patient remained in complete symptomatic remission and had achieved full range of motion at the sixth month of re-evaluation. Active cervical range of motion was restored to 90°/90° rotation, 80°/80° flexion, and 50°/70° extension. Her WHOQOL score improved from 64 to 98. Follow-up radiography revealed normal cervical lordosis (Figure 2B). The patient was enrolled in elective maintenance care with monthly scheduled visits for monitoring, manipulation, home exercises, ergonomic counseling, and lifestyle advice change, including reducing the time hours of her mobile device.

## Discussion

The cervical spine is a highly complex structure that maintains the position of the head while allowing head movement. It also provides stability and protects the spinal cord [9,10]. The cervical spine anatomy dictates the degree of physiological motion at each level [9]. The cervical mechanoreceptor input provides important sensory feedback that contributes to the maintenance of normal postural balance. Cervical proprioception deficiency can result in severe dizziness [10]. The cervicogenic proprioceptive mechanism can be impacted by several neck disorders, including muscular exhaustion, cervical spondylosis, and whiplash injury [10]. Vertebrobasilar artery insufficiency, agitation of the cervical sympathetic nervous system, and abnormal pain signals from the upper cervical spine can also lead to a sensation of disequilibrium [11].

Self-manipulation or "cracking" of the neck may provide temporary relief from stiffness or pain as the joint motion is temporarily moved, but it poses a significant risk of serious harm. Self-manipulation can generate a "long lever effect" with excessive and unbalanced force. The sharp movements during the uncontrolled self-manipulation can result in whiplash injury, which leads to additional neurological/spinal tract signs. Twisting and forcing the neck can shift the head's center of gravity, causing an increase in cantilever loads. This can be especially harmful to the higher cervical joints [12]. Excessive neck bending also causes stretching of the cervical spine and other spinal components. The improper head position has been linked to a wide range of disorders, including CGD and vertigo, cervical radiculopathy, and cervicogenic headache [12]. Most of these disorders present as combinations of uncomfortable symptoms and spinal abnormalities [12]. In addition, damage to the vertebrae, discs, and ligaments leads to conditions, such as osteoarthritis, herniated discs, and spinal instability [4-7]. It could also lead to a direct injury of the spinal cord or nerve roots, resulting in sensory and motor deficits [4-7].

There is an increased risk of developing these complications, in the presence of preexisting cervical conditions, especially if manipulation is performed incorrectly or with excessive force [13]. As the average person lacks advanced anatomical knowledge and proper training in manual techniques, self-manipulation of the cervical spine is not advisable. The self-manipulation creates a "long lever effect," which generates forces that are unbalanced and excessive. The strong/sharp movements during uncoordinated manipulation can result in a whiplash type of injury, which lead to muscular or neurological damage. Complex spinal manipulations should only be performed by a licensed health professional with experience in assessing cervical dysfunction and competence in performing specific adjustments and mobilization procedures. By seeking treatment from a trained chiropractor, patients can gain relief from neck pain and stiffness while ensuring that the safest and most effective manipulation or mobilization approach is practiced [14]. A chiropractor can also conduct a detailed assessment, identify the underlying causes of pain, stiffness, or limited range of motion, and ultimately prescribe a treatment regimen based on appropriate evidence-based techniques using a controlled force and precise patient positioning [15]. Chiropractors also have the expertise to recognize any contraindications to the treatment regimen or signs of injury that may result from manipulation [16].

Hence, while self-manipulation of the cervical spine may provide temporary relief, it can easily lead to serious trauma and permanent damage in the long term [4-7]. Chiropractic care, including spinal manipulation therapy and rehabilitative exercises, is effective in the management of CGD [17,18]. Spinal manipulation can help reduce pain and improve balance in patients with cervical spine disorders [2]. As this case illustrates, self-manipulation of the cervical spine can lead to acute symptoms requiring professional treatment. Greater awareness is required regarding the dangers of cervical spine self-manipulation. Patients should be counseled to seek appropriate specialized care for cervical dysfunction or injury. More reports of CGD may highlight the prevalence of self-manipulation of the cervical spine. Although the nature of the case report lacks generalizability, follow-up, bias, diagnostic details, and adverse event analysis, the impact of related consequences can spur improved education and awareness regarding neck health and the appropriate time to consult a chiropractic doctor or other specialists.

## Conclusions

This case report highlights the risks associated with self-manipulation of the cervical spine and the effectiveness of chiropractic care in the management of CGD. Patients should be counseled that self-manipulation of the cervical spine can lead to joint and ligament injuries, disc herniation, and nerve trauma. While it provides temporary pain relief, it is associated with a high risk of potentially permanent damage. Conservative chiropractic treatment for dizziness arising from cervical spine dysfunction includes spinal manipulation and rehabilitative exercises. This offers a safe and comprehensive approach to diagnosis and management. Chiropractic care can successfully help alleviate CGD and improve patients' quality of life by restoring joint mobility and proper neck mechanics and enhancing neuromuscular control. Hence, patients should be counseled to seek evaluation and treatment from appropriate medical professionals for neck issues or dizziness/imbalance, rather than practicing self-manipulation of the cervical spine.
